# The occurrence of clubroot in cruciferous crops correlates with the chemical and microbial characteristics of soils

**DOI:** 10.3389/fmicb.2023.1293360

**Published:** 2024-01-08

**Authors:** Huajun Kang, Zihan Lin, Xiaowei Yuan, Yanxia Shi, Xuewen Xie, Lei Li, Tengfei Fan, Baoju Li, Ali Chai

**Affiliations:** ^1^Institute of Vegetables and Flowers, Chinese Academy of Agricultural Sciences, Beijing, China; ^2^College of Horticulture, Gansu Agricultural University, Lanzhou, China; ^3^Huasheng Seed Group Co., Ltd, Qingzhou, China

**Keywords:** clubroot, suppressive soil, soil chemical properties, microbial community composition, microbial network

## Abstract

Clubroot disease, caused by *Plasmodiophora brassicae*, is a serious soil-borne disease in Brassica crops worldwide. It seriously occurs in conducive soils of southern China, while never happens in some areas of northern China with suppressive soils. To understanding the differences, we measured the soil suppressiveness, chemical properties, and microbial communities in suppressive and conducive soils by bioassay and sequencing of 16S and 18S rRNA amplicons. The biological basis of clubroot suppressiveness was supported by the ability to remove it by pasteurization. The pH value and calcium content in the suppressive soils were higher than those in the conducive soils. Suppressive soils were associated with higher fungal diversity and bacterial abundance. The fungal phyla Chytridiomycota, Olpidiomycota, and Mucoromycota and the bacterial phyla Acidobacteriota and Gemmatimonadota were enriched in suppressive soils. More abundant beneficial microbes, including *Chaetomium* and *Lysobacter*, were found in the suppressive soils than in the conducive soils. Molecular ecological network analysis revealed that the fungal network of suppressive soils was more complex than that of conducive soils. Our results indicate that plant health is closely related to soil physicochemical and biological properties. This study is of great significance for developing strategies for clubtroot disease prevention and control.

## 1 Introduction

Many crops are attacked by soil-borne pathogens worldwide, and the resulting diseases are difficult to control due to the complexity of the soil. Clubroot, which is caused by *Plasmodiophora brassicae*, is an important disease of plants of the family Brassicaceae (Dixon, 2009a). The disease is found throughout the world wherever crucifers (e.g., *Brassica napus*) are grown (Wallenhammar, 1996; Donald and Porter, 2009; Feng et al., 2013) and causes 10−15% of yield losses worldwide (Dixon, 2009b; Faggian and Strelkov, 2009). In China, clubroot disease has occurred in many provinces except Shānxi, Qinghai Province, Ningxia Hui Autonomous Region, and Inner Mongolia Autonomous Region. Clubroot infection is much more severe in Shandong, Liaoning, Jilin, Shānxi, Jiangxi, Yunnan, Sichuan, Guizhou, Chongqing, Hunan, Hubei Provinces, and the Tibet Autonomous Region of China (Supplementary Figure 1). Field studies have indicated that the pathogen has a half-life of at least 3.6 years, and some spores may exist for at least 17.3 years in soil in the absence of suitable hosts before spore populations are eroded to undetectable levels (Wallenhamar, 2010). Although many methods have been proposed, it is still difficult to control the disease (Niwa et al., 2008).

Plant diseases are determined by the interactions among host, pathogen, and the environment. It is the balance of these interactions among three elements that determines whether or not disease develops to destructive levels in a particular situation (Keane and Kerr, 1997). Among them, environmental factors, including biotic and abiotic factors have been considered to play an important role in the development of plant diseases, especially for soil-borne diseases (Keane and Kerr, 1997; Husson et al., 2021). Several studies have confirmed that soil abiotic factors can influence these interactions and thereby disease incidence or severity (Nerey et al., 2010; Tamm et al., 2010; Mehmood et al., 2013; You et al., 2017). For instance, soil pH and calcium showed a significantly negative correlation with the wilt infection rate (Yang et al., 2017). For root rot, Calcisol is always the most disease-conducive soil, and Vertisol is the most disease-repressive soil (Nerey et al., 2010). For clubroot, soil type, pH, and calcium, boron and nitrogen concentrations are known to have substantial effects on the infection and development of *P*. *brassicae*, but specific information on these factors is often surprisingly weak and incomplete (Gossen et al., 2014). Root hair infection and subsequent symptom development were limited when the soil pH was higher than 7.2, but infection of root hairs was not affected by high pH in the absence of calcium (Myers and Campbell, 1985; Rashid et al., 2013). Previous studies have shown that boron slows the development of *P*. *brassicae* during infection of root hairs and the root cortex, and the effect of boron on clubroot was related to soil type (Deora et al., 2011, 2014).

Moreover, soil and plant health also depend largely on the composition and diversity of the soil microbial community (van Elsas et al., 2012). Different microbial species in soil microbial communities have complex interactions (such as symbioses, parasitism, competition, or predation), forming a complex interrelated ecological network (Poudel et al., 2016). For example, healthy soils contained higher microbial diversity and more abundant beneficial microbes than bacterial wilt-infected soils (Wang et al., 2017). A higher abundance of *Fusarium* was observed in *Fusarium* wilt-diseased soils, whereas the phylum Firmicutes and the genera *Bacillus*, *Lactococcus*, and *Pseudomonas* were enriched in disease-free soils (Zhou et al., 2019). The healthy soil network contained more interacting species, more key microorganisms, and better high-order organization than bacterial wilt-susceptible soil (Qi et al., 2019). A recent study found that initial soil microbiome composition and functioning predetermine future plant health (Wei et al., 2019).

Disease suppressive soils have been defined as those in which disease development is minimal even in the presence of a virulent pathogen and a susceptible host (Mazzola, 2010). Classically, soil suppressiveness has been considered either general or specific (Janvier et al., 2007). General suppression is linked with the total microbial biomass in soil or related to physical or chemical attributes of the soil (Weller et al., 2002; Mazzola, 2010). Specific suppression is superimposed over the background of general suppression and is due, at least in part, to the effects of individual or select groups of microorganisms during some stage in the life cycle of a pathogen (Weller et al., 2002). The biotic factors that contribute to specific soil suppressiveness have been elucidated for a number of plant-pathogen systems (Rosenzweig et al., 2012; Penton et al., 2014; Giné et al., 2016; Lee et al., 2021). The soil suppressiveness of clubroot disease has been studied, and it has been suggested that both abiotic and biotic factors play an important role in disease suppression (Murakami et al., 2000). However, the differences in soil properties, microbial community composition and structure, and microbial networks between clubroot-suppressive and clubroot-conducive soils are still unclear.

In this study, we hypothesized that the chemical properties, microbial community and structure, and microbial network of suppressive soils are different from those of conducive soils. Therefore, the objectives of this study were to (1) compare the chemical properties between suppressive soils and conducive soils, (2) determine the relationships between soil microbial properties and plant health, and (3) investigate key microorganisms associated with plant health in networks. This study will present novel insight into understanding the differences of soil characteristics and microbiota between clubroot-suppressive and conducive soils, which will contribute to the development of new strategies for the control of clubroot disease.

## 2 Materials and methods

### 2.1 Site description and sample collection

Soil samples were collected from ten different sites (Supplementary Figure 1) with cultivated cruciferous vegetables, and detailed information is listed in Table 1. Among the 10 sites, five sites (Xinmin City, Liaoning Province; Licang District, Shandong Province; Jianshi County, Hubei Province; Lichuan City, Hubei Province; Youxian District, Sichuan Province) had a clubroot infection rate of more than 70%. The soil samples, with infestation levels of approximately 10^5^−10^8^ spores⋅g^–1^ were determined to be conducive soils, and classified into two soil types Luvisols and Cambisols. Clubroot disease was not observed in the other five sites (Yuci District, Shānxi Province; Xia County, Shānxi Province; Yuanzhou District, Ningxia Hui Autonomous Region; Shule County, Xinjiang Uygur Autonomous Region; Linhe District, Inner Mongolia Autonomous Region), and the soils were confirmed to be free of *P. brassicae* by qPCR and tentatively classified as suppressive soils, which were classified into Cambisols, Luvisols, and Kastanozems.

**TABLE 1:** Description of soil samples.

Sample location	Clubroot infection,%	Resting spores/g soil	Coordinates	Altitude (m)	Mean annual temperature(°C)	Mean annual precipitation (mm)	Year	Soil type	Crops
Yuci District, Shānxi Province	0.00	0.00	37.5767° N 112.7338° E	768	19.0	193.4	2020	Luvisols	Cabbage
Xia County, Shānxi Province	0.00	0.00	35.0522° N 111.1554° E	180	13.1	539.5	2020	Cambisols	Chinese cabbage
Yuanzhou District, Ningxia Hui Autonomous Region	0.00	0.00	36.0794° N 106.1724° E	1705	6.1	492.2	2020	Cambisols	Chinese cabbage
Linhe District, Inner Mongolia Autonomous Region	0.00	0.00	40.8080° N 107.3157° E	1055	7.4	150.3	2020	Kastanozems	Chinese cabbage
Shule County, Xinjiang Uygur Autonomous Region	0.00	0.00	39.2279° N 76.3321° E	1250	11.8	66.1	2020	Cambisols	Chinese cabbage
Xinmin City, Liaoning Province	80.26	7.99 × 10^7^	41.8705° N 122.9656° E	35	8.6	600.0	2020	Luvisols	Chinese cabbage
Licang District, Shandong Province	75.38	8.60 × 10^6^	36.1511° N 120.4267° E	99	14.5	732.0	2020	Luvisols	Chinese cabbage
Lichuan City, Hubei Province	77.46	2.00 × 10^5^	30.3212° N 108.6638° E	1595	12.3	1200.0	2020	Cambisols	Chinese cabbage
Jianshi County, Hubei Province	72.29	9.00 × 10^6^	30.7344° N 109.7990° E	726	16.0	1480.0	2020	Cambisols	Chinese cabbage
Youxian District, Sichuan Province	85.73	1.15 × 10^8^	31.5959° N 104.8624° E	568	16.4	969.6	2020	Cambisols	Cabbage

For each site, 3 random subplots (approximately 60 m^2^) were chosen, and soil samples from approximately 10 healthy (suppressive soil) or 10 clubroot-diseased (conducive soil) plants from each subplot were collected using the checkerboard sampling method during August 2020. Briefly, each subplot was divided into 10 areas and rhizosphere soil of plant in the central point of each area was collected (Niu et al., 2017). The 10 soil samples from each subplot were mixed to form one composite sample. The composite samples were placed into sterile bags and transported to the laboratory at 0°C. Samples were then divided into two subsamples: one portion (50 g) was stored at −80°C for DNA extraction, and another (300 g) was used for chemical property analysis after thorough homogenization through a 2-mm sieve and air-drying. The soil (200 kg) used for the pot experiments was collected with a shovel from each subplot and thoroughly mixed for each site.

### 2.2 Quantification of *P*. *brassicae* in soils

For each composite soil, total DNA was extracted from 0.5 g of soil using the FastDNA^®^ Spin kit (MO BIO Laboratories, Inc., Carlsbad, CA, USA) according to the manufacturer’s instructions. The abundance of *P*. *brassicae* in the soil was quantified using quantitative polymerase chain reaction (qPCR) according to established methods (Chai et al., 2016). Fluorescence was detected after each cycle, and the concentration of pathogen was determined via qPCR according to the protocol described in our previous work (Chai et al., 2016).

### 2.3 Bioassay to assess the disease suppressiveness of the soils

To determine the suppressiveness of the soils, pot experiments were performed based on Mendes et al. (2011) with some modifications in a greenhouse in Qingzhou City, Weifang City, Shandong Province, in September 2020. In brief, three treatments were compared: (i) natural soil without the addition of *P*. *brassicae*, (ii) natural soil with the addition of *P*. *brassicae*, and (iii) pasteurized soil (80°C for 1 h) with the addition of *P*. *brassicae*. All 10 soil samples were used for pot experiments.

*P*. *brassicae*-infected Chinese cabbage samples were collected from a natural field in Beijing and stored at −20°C. The resting spores were prepared by the method of Castlebury et al. (1994). The soils were sprayed with suspensions of resting spores (25 mL kg^–1^ soil) and mixed thoroughly to obtain 10^8^ resting spores g^–1^ soil.

Chinese cabbage (cv. Sushenglvxiu) seeds were sown in sterilized plastic pots (19 cm × 14 cm × 11.5 cm). Four seedlings were planted in each pot. Treatments were arranged in a randomized complete block design and replicated three times with five pots serving as a replicate. Plants were incubated in a greenhouse (25°C; 80% relative humidity and 16 h light and 8 h dark). Roots of each Chinese cabbage plant were removed 35−40 days after sowing and examined for gall formation. Disease severity was scored according to the following classes (Wallenhammar et al., 2012): 0, no galls; 1, enlarged lateral roots; 2, enlarged taproot; 3, enlarged napiform taproot, lateral roots healthy; and 4, enlarged napiform taproot, lateral roots infected. The disease severity index (DSI) was calculated according to the method of Chai et al. (2016). The experiments were independently repeated three times.

### 2.4 Soil chemical analysis

Electrical conductivity (EC) was measured using a conductivity meter in a soil water suspension (1:5 w/v) after shaking for 30 min. The soil pH was determined with a pH meter (Shanghai Sanxin Instrumentation, Inc., China) in a 1:5 (m/m) soil to water ratio, and the suspension was shaken at 200 r min^–1^ for 15 min at 25°C. The cation exchange capacity (CEC) was measured by the method of Mulvaney et al. (2004). Available K (AK) was extracted with ammonium acetate and determined by flame photometry using an FP640 Flame Photometer (Shanghai Instruments Group Co., Ltd., China). Available phosphorus (AP) was determined using the sodium hydrogen carbonate solution-Mo-Sb anti-spectrophotometric method (Shen et al., 2011). Soil organic matter (SOM) and available N (AN) were determined using the potassium dichromate external heating method (Ciavatta et al., 1991) and the alkaline-hydrolyzable diffusion method (Li et al., 2014), respectively. The total nitrogen (TN) level was determined using the semimicro Kjeldahl method. The exchangeable calcium (Ca) was measured by the method of Bao (2000). The available boron (B) level was determined as previously described (Bhering et al., 2017).

### 2.5 Amplicon sequencing and data processing

Total DNA of soil was extracted using a PowerSoil DNA Isolation Kit (MoBio Laboratories, Carlsbad, CA) according to the manufacturer’s protocols. Each composite soil sample was extracted in triplicate, and the extracted DNA solutions were pooled. The primer set ITS1F (5′-CTTGGTCATTTAGAGGAAGTAA-3′) (Gardes and Bruns, 1993) and ITS2 (5′-GCTGCGTTCT TCATCGATGC-3′) was selected to target the fungal ITS1 region. 338F (5′-ACTCCTACGGGAGGCAGCAG-3′) and 806R (5′-GGACTACNNGGGTATCTAAT-3′) were used to amplify the V4 hypervariable regions of the bacterial 16S rRNA gene. Amplicons were sequenced on the MiSeq platform at Allwegene CO., LIMITED (Beijing, China).

To obtain high-quality sequences, quality filtering of the raw sequences was performed using the QIIME package (Quantitative Insights Into Microbial Ecology) (v1.2.1). After detecting and removing the chimeric sequences using UCHIME V4.2, the effective sequences were obtained and used to perform operational taxonomic unit (OTU) and species annotation. Qualified reads were clustered into operational taxonomic units (OTUs) at a similarity level of 97% using the Uparse algorithm of Vsearch (v2.7.1) software (Edgar, 2013). QIIME (v1.8.0) was used to calculate the richness and diversity indices based on the OTU information. Sequences were compared to the entries in the SILVA database (Release 128/132/138,^1^ bacteria) and Unite database (Release 8.2,^2^ fungi) with a confidence threshold of 80% and phylogenetically assigned to taxonomic classifications using the Ribosomal Database Project naïve Bayesian classifier^3^ at a distance of 0.03.

### 2.6 Construction and analysis of the microbial network

We used the phylogenetic molecular ecological network (pMEN) method to construct interaction networks for disease-suppressive and disease-conducive soils (Deng et al., 2012; Qi et al., 2019). Random matrix theory (RMT) was used to automatically identify the appropriate similarity threshold prior to network construction. All analyses were performed using the Molecular Ecological Network Analyses Pipeline,^4^ and network graphs were visualized using Cytoscape 2.8.2 software (Su et al., 2014).

### 2.7 Statistical analysis

Soil physicochemical characteristics, Chinese cabbage clubroot DSI, and alpha diversity indices between suppressive and conducive soils were compared using Tukey’s HSD multiple range test. A Tukey HSD test was performed using SPSS Statistics 20.0 software (IBM, New York, USA). To examine the similarity between different samples, partial least squares discrimination analysis was performed by R (v3.6.0) (R Core Team, 2018) based on the OTU information. Redundancy analysis (RDA) using the vegan package in R version 3.6.0 was performed to analyze the relationships between microbial community structure and environmental variables. The significant differences in bacterial and fungal species between suppressive and conducive soils were compared using the Wilcoxon rank-sum test (*P* < 0.05) in R version 3.6.0. The linear discriminant analysis (LDA) effect size (LEfSe) method (LDA score > 3.0, *P* < 0.05) was used to analyse the association of the top 50 abundant fungal and bacterial genera with the disease-suppressive and -conducive soils.

## 3 Results

### 3.1 *P*. *brassicae* abundance at different sites

The abundance of *P*. *brassicae* was analyzed in each soil sample, and the results are presented in Table 1. *P. brassicae* was not detected in any suppressive soils. Quantifiable levels of *P. brassicae* were detected in all five conducive soil samples, ranging from 2.00 × 10^5^−1.15 × 10^8^ resting spores per g of soil. The highest level of resting spore infestation, 1.15 × 10^8^ resting spores per g of soil, was found in conducive soil collected from Youxian District.

### 3.2 Identification of soils suppressive to *P*. *brassicae*

A collection of 10 soils of diverse geographical origins was used for disease suppressiveness against *P*. *brassicae* in greenhouse pot experiments. No symptoms of clubroot disease were observed in plants planted to suppressive soils in treatment with or without the addition of *P*. *brassicae* (Table 2). The suppressive soils lost clubroot suppressiveness after sterilization (80°C 1 h), and the DSIs of plants grown in sterilized suppressive soil ranged from 56.25 ± 4.45−73.75 ± 4.45, which were lower than those of the conducive soils (*P* < 0.05; Tukey’s HSD). The plants exposed to each conducive soil always developed clubroot symptoms in all three treatments. Altogether, these results indicate that soil suppressiveness was correlated with soil microbial communities.

**TABLE 2:** Disease index of clubroot disease of Chinese cabbage in ten different agricultural soils.

Soil traits	Sample location	Disease index		
		**Natural soil without *P*. *brassicae***	**Natural soil with *P*. *brassicae***	**Sterile soil with *P*. *brassicae***
Suppressive	Yuci District, Shānxi Province	0.00 ± 0.00d	0.00 ± 0.00c	63.33 ± 1.52cd
	Xia County, Shānxi Province	0.00 ± 0.00d	0.00 ± 0.00c	56.25 ± 4.45d
	Yuanzhou District, Ningxia Hui Autonomous Region	0.00 ± 0.00d	0.00 ± 0.00c	67.92 ± 2.12bc
	Linhe District, Inner Mongolia Autonomous Region	0.00 ± 0.00d	0.00 ± 0.00c	73.75 ± 4.45b
	Shule County, Xinjiang Uygur Autonomous Region	0.00 ± 0.00d	0.00 ± 0.00c	68.75 ± 4.68bc
Conducive	Xinmin City, Liaoning Province	93.33 ± 4.71a	96.67 ± 2.36a	91.67 ± 2.57a
	Licang District, Shandong Province	88.93 ± 4.29a	99.17 ± 1.18a	93.75 ± 2.70a
	Lichuan City, Hubei Province	7.50 ± 2.04d	76.67 ± 4.12b	92.92 ± 2.12a
	Jianshi County, Hubei Province	22.92 ± 4.12c	97.08 ± 2.12a	90.83 ± 1.18a
	Youxian District, Sichuan Province	72.92 ± 3.58b	92.15 ± 4.55a	90.00 ± 2.04a
	Control	0.00 ± 0.00d	98.75 ± 1.02a	92.92 ± 4.12a

Values are means ± standard deviation. Means followed by a different lower case letter for a given factor are significantly different (*P* < 0.05; Tukey’s HSD).

### 3.3 Soil chemical properties

The soil pH values of all the suppressive soils were greater than ≥8.16 and higher than those of the conducive soils (*P* < 0.05; Tukey’s HSD). Among the conducive soils, the pH of soil collected from Xinmin City and Licang District was significantly higher than that of soil collected from Lichuan City, Jianshi County and Youxian District (*P* < 0.05; Tukey’s HSD). Similar to pH, the contents of exchangeable calcium in the suppressive soils were significantly higher than those in the conducive soils (*P* < 0.05; Tukey’s HSD). No significant association was observed for SOM, TN, AN, AP, AK, CEC, EC, and B with the presence of the pathogen or with the suppressive phenotype (Table 3). The above results indicated that the occurrence of clubroot may be related to soil pH and available calcium content.

**TABLE 3:** Soil properties at 10 sampling districts.

Soil traits	Sample location	pH	SOM (g/kg)	TN (g/kg)	AN (mg/kg)	AP (mg/kg)	AK (mg/kg)	CEC (cmol (+)/kg)	EC (ms/cm)	Ca (g/kg)	B (mg/kg)
Suppressive	Yuci District, Shānxi Province	8.48 ± 0.03b	16.14 ± 0.54e	0.96 ± 0.00f	63.93 ± 2.88f	45.97 ± 0.73f	111.67 ± 0.47f	12.07 ± 0.41bc	214.00 ± 0.82e	7.05 ± 0.09d	0.45 ± 0.04de
	Xia County, Shānxi Province	8.83 ± 0.02a	16.40 ± 0.14e	1.14 ± 0.01e	114.33 ± 2.88de	100.52 ± 0.64c	344.17 ± 16.11b	16.90 ± 0.91a	949.00 ± 11.22a	11.97 ± 0.02a	0.75 ± 0.02c
	Yuanzhou District, Ningxia Hui Autonomous Region	8.30 ± 0.03c	13.58 ± 0.24g	0.86 ± 0.02g	45.27 ± 1.75g	23.58 ± 0.61g	183.00 ± 1.63e	11.27 ± 0.34c	227.33 ± 2.05e	9.36 ± 0.19b	0.31 ± 0.06de
	Linhe District, Inner Mongolia Autonomous Region	8.16 ± 0.04d	16.02 ± 0.93ef	0.95 ± 0.01f	76.53 ± 3.49f	83.93 ± 1.52d	218.33 ± 1.25d	9.00 ± 0.59d	388.33 ± 11.03c	8.27 ± 0.07c	1.20 ± 0.15b
	Shule County, Xinjiang Uygur Autonomous Region	8.57 ± 0.01b	30.37 ± 0.62b	1.72 ± 0.02b	144.67 ± 1.75c	80.86 ± 0.92e	127.50 ± 0.94f	14.10 ± 0.16b	908.00 ± 4.32b	11.67 ± 0.15a	1.49 ± 0.03a
Conducive	Xinmin City, Liaoning Province	8.15 ± 0.02d	20.38 ± 0.70d	1.38 ± 0.02d	115.27 ± 0.66d	82.54 ± 1.08de	260.50 ± 2.45c	17.57 ± 1.09a	392.67 ± 8.18c	4.69 ± 0.10e	0.47 ± 0.02d
	Licang District, Shandong Province	7.54 ± 0.00e	15.56 ± 0.79ef	0.82 ± 0.00g	102.20 ± 1.14e	97.61 ± 0.54c	120.83 ± 0.47f	6.57 ± 0.25e	330.67 ± 17.25d	2.36 ± 0.03g	0.34 ± 0.02de
	Lichuan City, Hubei Province	5.20 ± 0.03g	37.72 ± 0.93a	2.31 ± 0.04a	257.60 ± 1.14a	119.28 ± 0.85b	533.17 ± 7.76a	12.50 ± 1.02bc	66.36 ± 3.16f	2.26 ± 0.02g	0.25 ± 0.02e
	Jianshi County, Hubei Province	5.13 ± 0.02g	36.64 ± 1.19a	2.23 ± 0.02a	259.93 ± 9.86a	145.19 ± 0.58a	340.83 ± 2.05b	12.30 ± 0.33bc	25.20 ± 1.26g	1.82 ± 0.02h	0.35 ± 0.04de
	Youxian District, Sichuan Province	6.11 ± 0.04f	24.15 ± 0.38c	1.62 ± 0.03c	203.93 ± 0.66b	44.22 ± 0.55f	247.50 ± 2.83c	14.17 ± 0.34b	43.37 ± 4.29fg	3.90 ± 0.02f	0.39 ± 0.05de

Values are means ± standard deviation. Means followed by a different lower case letter for a given factor are significantly different (*P* < 0.05; Tukey’s HSD).

### 3.4 Microbial diversity composition and structure

#### 3.4.1 Fungal composition and structure

**FIGURE 1:**
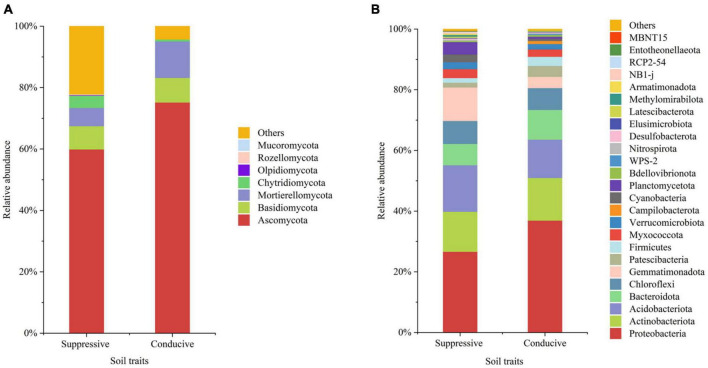
The average relative abundances in the top 20 fungal **(A)** and bacterial **(B)** phyla from disease-suppressive and disease-conducive soils. Others represent phyla of low relative abundance that rank lower than 20.

**FIGURE 2:**
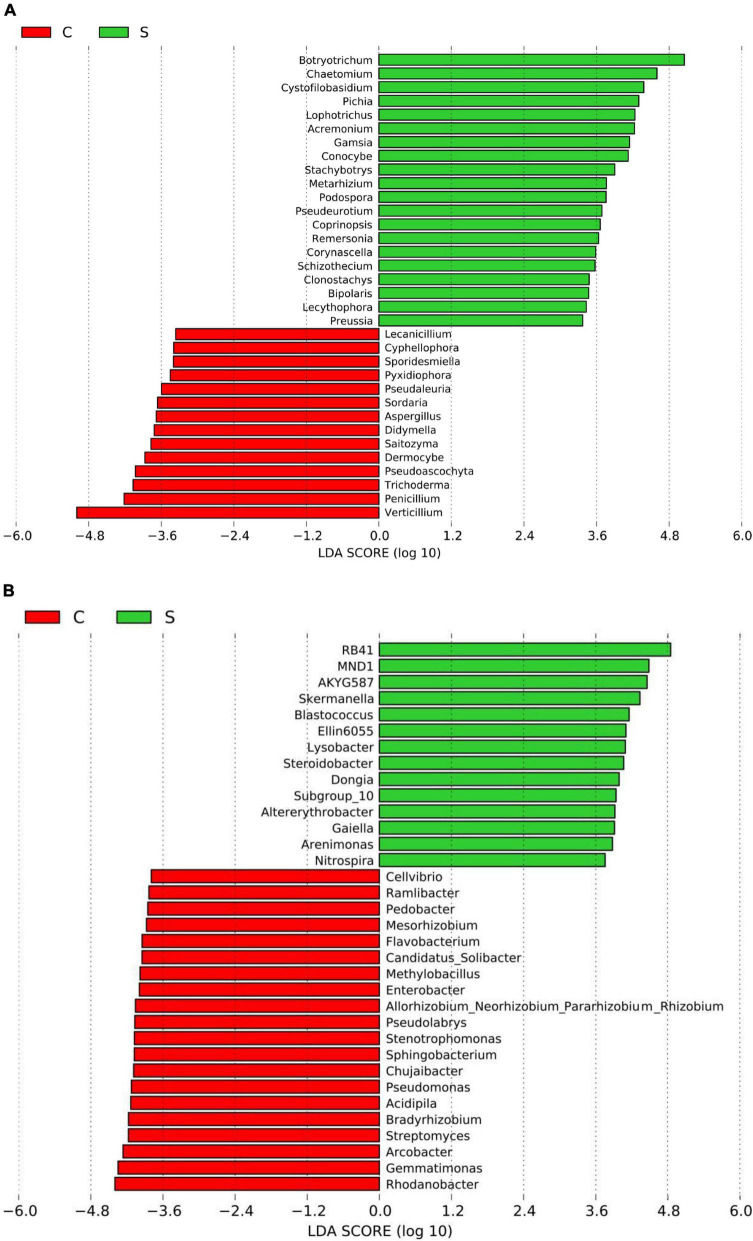
Histogram of the LDA scores computed for differentially abundant fungal **(A)** and bacterial **(B)** genera between the disease-suppressive and disease-conducive soils. C, conducive soil; S, suppressive soil.

A total of 4, 160, 237 reads were obtained from 10 soil samples, and 4, 962 OTUs were identified from the reads. The analysis of the estimated Shannon indices revealed that the suppressive soils harbored higher fungal diversity (*P* < 0.05; Tukey’s HSD), whereas the richness indices (Chao 1) and OTU numbers showed no significant difference between suppressive soils and conducive soils (Table 4). Fourteen fungal phyla were identified in all soil samples. Ascomycota was the most abundant phylum (67.45%), followed by Mortierellomycota (8.89%), Basidiomycota (7.80%), and Chytridiomycota (2.30%). The phylum Ascomycota was more abundant in conducive soils than in suppressive soils (*P* < 0.05; Tukey’s HSD), whereas the phyla Olpidiomycota, Chytridiomycota, and Mucoromycota showed the opposite trend (Figure 1A). The difference in fungal abundance between suppressive and conducive soils was analyzed by linear discriminant analysis. The fungal genera *Botryotrichum*, *Chaetomium*, *Cystofilobasidium*, *Pichia*, *Lophotrichus*, *Acremonium*, *Gamsia*, *Conocybe*, *Stachybotrys*, *Metarhizium*, *Podospora*, *Pseudeurotium*, *Coprinopsis*, *Remersonia*, *Corynascella*, *Schizothecium*, *Clonostachys*, *Bipolaris*, *Lecythophora*, and *Preussia* were more abundant in the suppressive soils than in the conducive soils. *Botryotrichum* was the most dominant genus, comprising 12.01% of the total fungal genera in suppressive soils, whereas *Verticillium* was the most dominant genus in conducive soils, accounting for 18.39% of the total fungal genera (only 0.06% in suppressive soils) (Figure 2A).

**TABLE 4:** OTUs, richness, and shannon indices of fungi and bacteria from different fields.

Soil traits	Sample location	Fungi			Bacteria		
		**OTUs**	**Chao 1**	**Shannon**	**OTUs**	**Chao1**	**Shannon**
Suppressive	Yuci District, Shānxi Province	537.00 ± 35.51bc	757.51 ± 57.98bc	5.83 ± 0.11a	3308.00 ± 95.44ab	4734.90 ± 174.56a	9.83 ± 0.07ab
	Xia County, Shānxi Province	508.33 ± 35.31cd	787.04 ± 83.21bc	4.60 ± 0.19bc	3496.67 ± 42.08a	4659.44 ± 114.97a	10.03 ± 0.02bc
	Yuanzhou District, Ningxia Hui Autonomous Region	668.33 ± 40.05ab	984.18 ± 31.34ab	6.01 ± 0.17a	3430.33 ± 28.71a	4824.53 ± 95.62a	9.92 ± 0.06ab
	Linhe District, Inner Mongolia Autonomous Region	545.00 ± 27.65bc	747.95 ± 58.00bc	5.10 ± 0.18ab	3350.33 ± 38.80a	4766.15 ± 128.07a	10.00 ± 0.04bc
	Shule County, Xinjiang Uygur Autonomous Region	628.67 ± 21.48ab	965.25 ± 31.76ab	5.47 ± 0.11ab	3613.33 ± 138.41a	5130.96 ± 124.15a	10.29 ± 0.03a
Conducive	Xinmin City, Liaoning Province	382.33 ± 18.26ef	656.29 ± 78.27d	3.98 ± 0.82cd	3285.33 ± 319.31ab	4630.65 ± 342.52a	9.57 ± 0.61bc
	Licang District, Shandong Province	345.33 ± 77.96f	542.96 ± 140.25d	2.25 ± 0.19e	2890.33 ± 25.53b	3778.71 ± 46.50b	9.63 ± 0.11ab
	Lichuan City, Hubei Province	434.00 ± 3.74de	681.43 ± 42.77cd	3.20 ± 0.12de	2346.67 ± 52.66c	3188.19 ± 96.23c	9.23 ± 0.03cd
	Jianshi County, Hubei Province	586.33 ± 56.07ab	904.36 ± 55.42ab	4.16 ± 0.56cd	2356.67 ± 76.93c	3491.89 ± 181.64bc	8.94 ± 0.09d
	Youxian District, Sichuan Province	700.00 ± 39.37a	1062.95 ± 36.73a	4.97 ± 0.20ab	3411.33 ± 44.89a	3875.45 ± 53.78b	9.34 ± 0.01bc

Values are means ± standard deviation. Means followed by a different lower case letter for a given factor are significantly different (*P* < 0.05; Tukey’s HSD).

**FIGURE 3:**
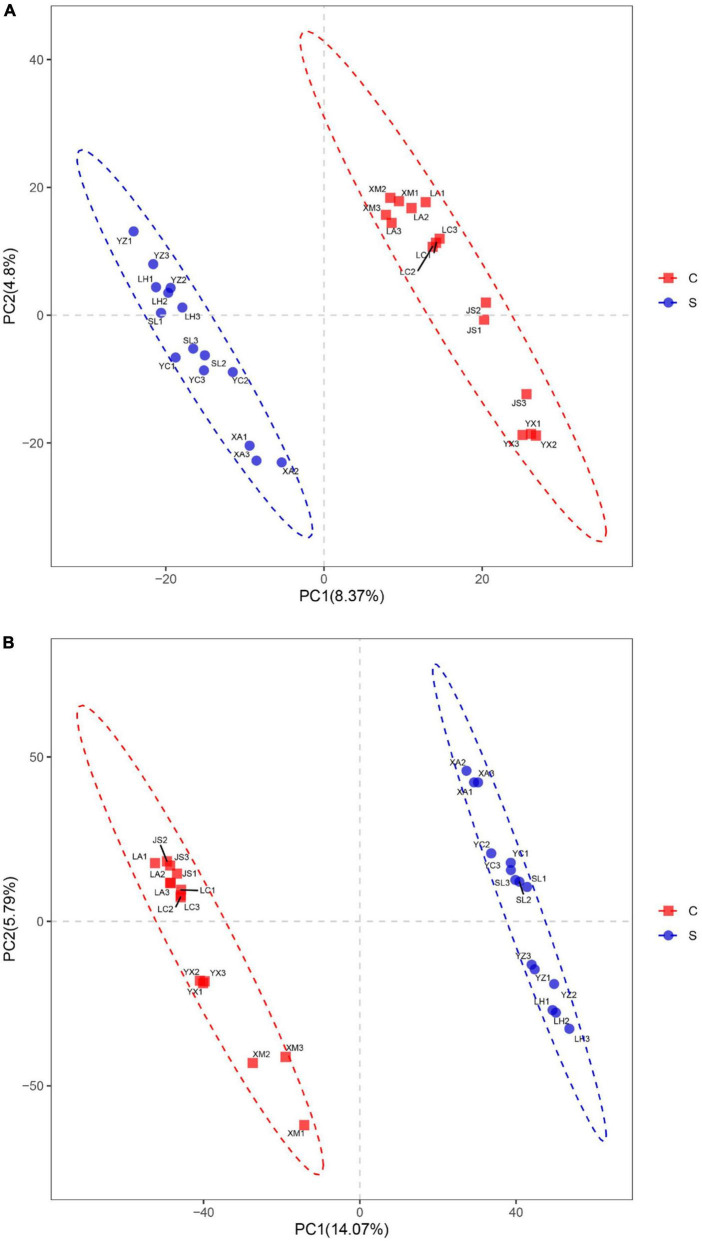
Partial least squares discrimination analysis of fungal **(A)** and bacterial **(B)** community structures in disease-suppressive and disease-conducive soils. C, conducive soil; S, suppressive soil. YC, Yuci District, Shānxi Province; XA, Xia County, Shānxi Province; YZ, Yuanzhou District, Ningxia Hui Autonomous Region; SL, Shule County, Xinjiang Uygur Autonomous Region; LH, Linhe District, Inner Mongolia Autonomous Region; XM, Xinmin City, Liaoning Province; LA, Licang District, Shandong Province; LC, Lichuan City, Hubei Province; JS, Jianshi County, Hubei Province; YX, Youxian District, Sichuan Province.

Partial least squares discrimination analysis (PLS-DA) was used to analyze the β-diversity of fungal communities. PC1 and PC2 represented 4.80 and 8.37%, respectively, of the variance, and the contribution of the cumulative variance of the two principal coordinates (PC1 and PC2) accounted for 13.17% (Figure 3A). The results showed that bacterial communities from suppressive soils were clearly separated from those from conducive soils.

#### 3.4.2 Bacterial composition and structure

A total of 2, 398, 352 reads were obtained from 10 soil samples, and 13, 650 OTUs were identified from the reads. The bacterial OTU number in the suppressive soils was higher than that in the conducive soils (*P* < 0.05; Tukey’s HSD), except for soil from Youxian District (Table 4). Analysis by Chao1 revealed a higher richness of bacteria in the suppressive soils. The Shannon index indicated that no significant difference was observed in the bacterial diversity of suppressive and conducive soils. In all soil samples, Proteobacteria, Acidobacteriota, Actinobacteriota, Bacteroidota, Chloroflexi, Gemmatimonadota, Myxococcota, Patescibacteria, Planctomycetota, Firmicutes, Verrucomicrobiota, and Cyanobacteria were the dominant phyla, accounting for 96.19% of the total bacterial sequences (Figure 1B). Acidobacteriota and Gemmatimonadota were more abundant in suppressive soils than in conducive soils (*P* < 0.05; Tukey’s HSD). The abundances of Proteobacteria, Patescibacteria, and Firmicutes were higher in conducive soils than in suppressive soils (*P* < 0.05; Tukey’s HSD). The LEfSe analytical method was used to visualize the representative taxa between suppressive and conducive soils. In the suppressive soils, the relative abundances of *RB41*, *MND1*, *AKYG587*, *Skermanella*, *Blastococcus*, *Ellin6055*, *Dongia*, *Lysobacter*, *Subgroup_10*, *Altererythrobacter*, *Gaiella*, *Arenimonas*, *Nitrospira*, and *Steroidobacter* were higher in suppressive soils than in conducive soils, whereas 19 genera were more abundant in conducive soils, such as *Rhodanobacter*, *Gemmatimonas*, and *Arcobacter* (Figure 2B).

The bacterial communities within the suppressive and conducive soil were separately clustered at the PC1 axis according to partial least squares discrimination analysis (PLS-DA). PC1 and PC2 explained 19.86% of the total bacterial community (Figure 3B).

### 3.5 Network analysis

**FIGURE 4:**
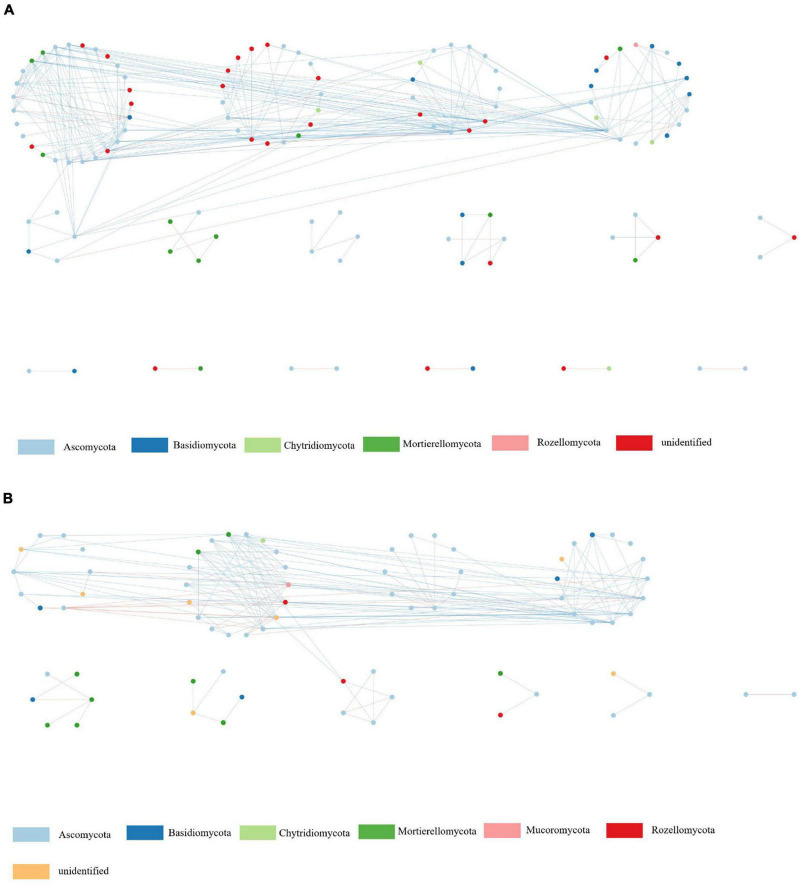
Network analyses of soil fungal communities in suppressive **(A)** and conducive **(B)** soil. Nodes of different colors belong to different bacterial phyla. Blue edges represent negative interactions between nodes. Red edges represent positive interactions.

In the fungal phylogenetic molecular ecological networks (pMENs), 120 and 76 nodes were constructed from suppressive (Figure 4A) and conducive (Figure 4B) soil samples, respectively. For suppressive and conducive networks, the average connectivity [Connectivity is the number of links (edges) of a node with other nodes] was 4.50 and 4.39, respectively. Suppressive and conducive soils harbored 8 and 6 modules with modularity values (Modularity measures the degree to which a network is organized into clearly delimited modules) of 0.44 and 0.41, respectively, (Table 5). Suppressive soils (270 links) had higher link numbers than conducive soils (167 links). With respect to bacteria, networks with 189 and 161 nodes were constructed from suppressive (Supplementary Figure 2A) and conducive (Supplementary Figure 2B) soil samples, respectively. The average connectivity was 2.77 and 9.44, and the average clustering coefficient value was 0.20 and 0.19 for suppressive and conducive networks, respectively, (Table 5). The suppressive and conducive soil networks had different modularity values (0.79 for suppressive and 0.25 for conducive soil). The total link numbers in suppressive and conducive soils were 262 and 760, respectively.

**TABLE 5:** Major topological properties of the empirical phylogenetic Molecular Ecological Networks (pMENs) of fungal and bacterial communities in disease-suppressive and disease-conducive soils and their associated random pMENs.

		Empirical networks						Random networks^b^		
	Soil trait	Similarity threshold	Network size (no. of nodes)^a^	Avg connectivity	Avg geodesic distance	Avg clustering coefficient	Modularity (no. of modules)	Avg geodesic distance	Avg clustering coefficient	Avg Modularity
Fungi	Suppressive	0.66	120	4.50	3.06	0.06	0.44 (8)	3.11 ± 0.08	0.10 ± 0.02	0.39 ± 0.01
	Conducive	0.77	76	4.39	3.52	0.07	0.41 (6)	2.97 ± 0.09	0.12 ± 0.02	0.37 ± 0.01
Bacteria	Suppressive	0.92	189	2.77	5.00	0.20	0.79 (12)	4.70 ± 0.13	0.02 ± 0.01	0.63 ± 0.01
	Conducive	0.74	161	9.44	3.06	0.19	0.25 (7)	2.63 ± 0.04	0.27 ± 0.02	0.21 ± 0.01

Values are means ± standard deviation.

a, The number of OTUs (nodes) in the network.

b, The random networks were generated by rewiring all of the links of a pMEN with the identical numbers of nodes and links to the corresponding empirical pMEN.

**FIGURE 5:**
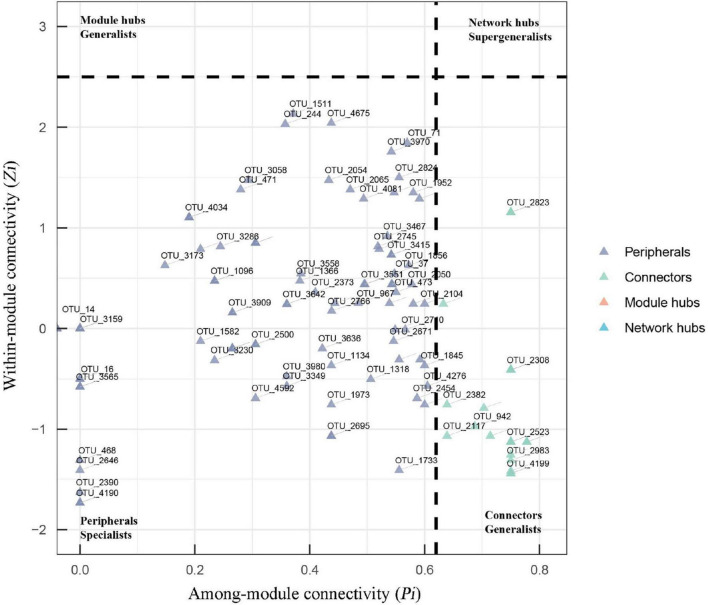
Zi-Pi plots showing the distribution of OTUs based on their topological roles in fungal networks. The threshold values of Zi and Pi for categorizing OTUs were 2.5 and 0.62, respectively. Nodes are defined as peripherals (Pi ≤ 0.62, Zi ≤ 2.5), module hubs (Pi ≤ 0.62, Zi > 2.5), connectors (Pi > 0.62, Zi ≤ 2.5) and network hubs (Pi > 0.62, Zi > 2.5).

The topological role of each node (OTU) was defined by the within-module connectivity (Zi) and among-module connectivity (Pi). In fungal networks, the majority of nodes (88.5%) were peripherals, while 11.5% were connectors (Figure 5 and Supplementary Table 1). No network hub or module hub was found in the fungal networks. More connectors (21) were found in the suppressive network than in the conducive network (19). Four connectors (OTU_2101, OTU_3841, OTU_403, and OTU_471) in the suppressive network are closely related to Ascomycota, Ascomycota, Olpidiomycota, and Chytridiomycota, respectively. The two connectors (OTU_4194 and OTU_4714) in the conducive network belonged to Mortierellomycota and Basidiomycota, respectively, (Supplementary Table 1). With respect to bacterial networks, the conducive network had more connectors (twenty) than the suppressive network (thirteen). In the conducive networks, OTU_10229, OTU_1140, OTU_1313, OTU_1477, and OTU_765 belonging to Proteobacteria and the other two connectors (OTU_7005 and OTU_9010) were closely related to Gemmatimonadota and Chloroflexi, respectively (Supplementary Figure 3 and Table 1).

### 3.6 Correlations between microbial communities and environmental factors

**FIGURE 6:**
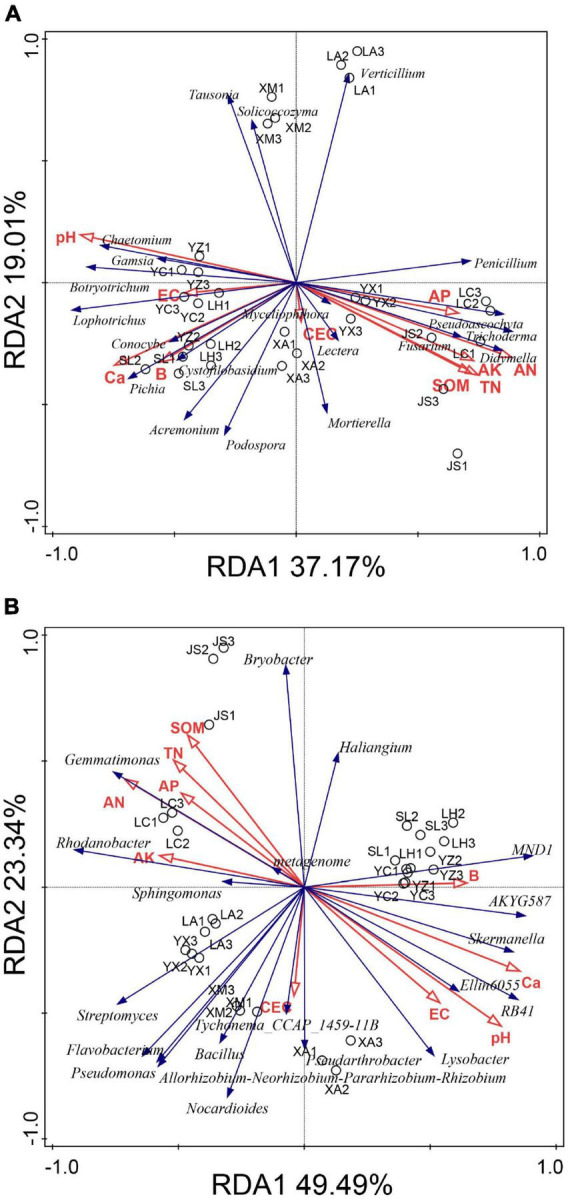
Redundancy analysis (RDA) of the fungal **(A)** and bacterial **(B)** communities based on genera distribution. YC, Yuci District, Shānxi Province; XA, Xia County, Shānxi Province; YZ, Yuanzhou District, Ningxia Hui Autonomous Region; SL, Shule County, Xinjiang Uygur Autonomous Region; LH, Linhe District, Inner Mongolia Autonomous Region; XM, Xinmin City, Liaoning Province; LA, Licang District, Shandong Province; LC, Lichuan City, Hubei Province; JS, Jianshi County, Hubei Province; YX, Youxian District, Sichuan Province.

The relationships between microbial community structure and soil chemical properties were analyzed with redundancy analysis (RDA) (Figure 6). The first and second RDA components explained 56.18 and 72.83% of the total fungal and bacterial variation, respectively. Redundancy analysis indicated that high abundances of *Chaetomium*, *Botryotrichum*, *Gamsia*, *Lophotrichus*, and *Acremonium* in the suppressive soils were positively correlated with pH, EC, Ca, and B but negatively correlated with AN, AP, AK, TN, and SOM. The relative abundances of *Fusarium*, *Penicillium*, *Pseudoascochyta*, *Trichoderma*, *Didymella*, *Lectera*, and *Mortierella* were positively correlated with AN, AP, AK, TN, and SOM but negatively correlated with pH, EC, Ca, and B (Figure 6A).

With respect to bacteria, SOM, TN, AN, AP, and AK were positively correlated with *Rhodanobacter*, *Gemmatimonas*, and *Sphingomonas* but negatively correlated with *Skermanella*, *Ellin6055*, *RB41*, and *Lysobacter* (Figure 6B).

## 4 Discussion

### 4.1 Soil suppressiveness is related to microorganisms in soil

Disease suppressive soils have been identified for many plant pathogens, and there is plenty of evidence for the role of both biotic and abiotic factors of the soil in disease suppression (Mazzola, 2002). Physical and chemical attributes of soil, such as texture and micronutrients, can operate in the suppression of plant diseases directly or indirectly through their impact on the activity of soil microorganisms (Mazzola, 2002). Although these abiotic characteristics of soil are beneficial to disease suppression, suppressiveness is fundamentally a function of microbiological activity of soil microorganisms or microbial metabolites. Higher abundances and diversity of microbes that harbor the non-ribosomal peptide (NRPS) gene were observed in *Fusarium* wilt disease-suppressive soil samples than in *Fusarium* wilt disease-conducive soil samples (Zhao et al., 2020). The study of *Fusarium* wilt-suppressive soils from Korea indicated that the suppressiveness has been attributed mainly to natural antibiotics secreted by *Streptomyces* sp. S4-7. In the present study, severe symptoms of clubroot were observed in plants exposed to sterilized suppressive soils, which indicated that the suppression of soil was related to soil microorganisms. This result follows the results of previous reports (Mendes et al., 2011; Cha et al., 2016; Xiong et al., 2017). However, the DSIs of plants grown in pasteurized suppressive soils were lower than those grown in control soil, which inferred that abiotic factors may also be critical in the suppressiveness of soils. Severe symptoms of clubroot were consistently observed on plants exposed to conducive soils, suggesting that soil biotic and abiotic factors were conducive to the occurrence of the disease, which is supported by previous studies (Murakami et al., 2000). Given that 80°C cannot completely eliminate all microorganisms, the soil microbiome left after heat treatment may still have pathogen inhibition.

### 4.2 Influence of soil chemical properties on plant disease

Various soil chemical properties not only affect pathogen growth and survival in soils but also influence the growth and health of plants. It has been reported that labile carbon is important for the maintenance of an abundant soil microbial community and the expression of soil suppressiveness (Bongiorno et al., 2019). Previous studies have shown that higher pH and AP may enhance the inhibition of banana *Fusarium* wilt disease. Reduction in root hair infection and subsequent reduction in clubroot development in brassicas at alkaline pH has been reported in some crops (Webster and Dixon, 1991a; Donald and Porter, 2004). Application of calcium, magnesium or boron to raise soil pH above 7.2 under controlled conditions reduced root hair infection and subsequent symptom development (Webster and Dixon, 1991a,b; Rashid et al., 2013). However, the pattern of low clubroot at neutral or slightly alkaline pH is not consistently observed in field situations (Karling, 1968; McDonald et al., 2004). In this study, all five suppressive soils were alkaline and had a higher pH than conducive soils. Consequently, higher pH values in soils probably indicate a healthier plant status. Notably, the pH values of the conducive soils collected from the Xinming and Licang sites were greater than 7.2, which may indicate that slightly alkaline soil pH alone did not eliminate the risk of clubroot when other conditions were conducive to pathogen infection and development. Boron application in brassica vegetables generally reduced clubroot incidence by up to 50% and increased yield by 30% (Dixon, 1996; Nott et al., 1999; Ruaro et al., 2009). However, there was no significant correlation between boron content and clubroot severity in this study. High concentrations of calcium in suppressive soils may suppress the occurrence of clubroot, which was in line with previous reports (Webster and Dixon, 1991b; Donald and Porter, 2004). Many studies have indicated that plant disease incidence is negatively correlated with the soil TON, AP, and AK contents (Oyarzun et al., 1998; Shen et al., 2014), but there were no significant correlations between these chemical properties and plant health in this study.

Based on the results of RDA, the beneficial microorganisms (e.g., *Chaetomium*, *Lysobacter*, and *RB41*) were positively correlated with the soil pH and Ca. These findings indicated that soil pH or calcium may enhance potential functional taxa to suppress clubroot.

### 4.3 Correlation between plant disease and soil microbial diversity or community structure

Soil microorganisms have profound effects on the growth, nutrition and health of plants in natural and agricultural ecosystems (Garbeva et al., 2004; Almario et al., 2013). The diversity and composition of the rhizosphere microbial community is a function of both plant species and soil properties (Ling et al., 2022). Previous investigations have shown that disruption of protective rhizobacteria abundance in the tomato rhizosphere led to the occurrence of bacterial wilt disease (Lee et al., 2021). The suppressive soils harbored a higher fungal abundance than the conducive soils in this study, which is consistent with the study of Wang et al. (2019). The bacterial diversity was higher in suppressive soils than in conducive soils, which is in line with the findings of Wang et al. (2017) and Rosenzweig et al. (2012) but in contrast with those of Xiong et al. (2017). Many studies have demonstrated that a diverse microbial community is often less prone to pathogen invasion than a simpler microbial community (Fargione and Tilman, 2005; van Elsas et al., 2012; Bonanomi et al., 2014; Chen et al., 2019). However, Meng et al. (2019) proposed that microbial diversity and abundance might not be consistent indicators for soil-supporting plant health. Consequently, the importance of soil microbial diversity varies in different systems.

Soil community structure is as important as community diversity in affecting plant health and the invasion of pathogens (Garbeva et al., 2004; Janvier et al., 2007). Liu et al. (2021) found that the fungal community outperformed the bacterial community in predicting plant health status. The occurrence of potato common scab has been reported to be related to community composition (Shi et al., 2019). The microbial community composition and structure were distinct between the suppressive soil and conducive soil in the present study. Ascomycota was more abundant in conducive soils than in suppressive soils, which was in agreement with a previous study (Xiong et al., 2017). The phyla Acidobacteriota and Gemmatimonadota were more abundant in our suppressive soils and are known to produce high levels of secondary metabolites (Crits-Christoph et al., 2018). This result was in agreement with the results of a previous study in which Acidobacteriota and Gemmatimonadota were more abundant in all *Fusarium* wilt-suppressive soils (Cha et al., 2016). Previous studies have shown that the relative abundance of several bacterial taxa was a more important indicator of disease suppression than the exclusive presence of specific bacterial taxa (Mendes et al., 2011; Cha et al., 2016). Our results suggested that members of these phyla may collectively play an important role in clubroot disease suppression.

The linear discriminant analysis (LDA) effect size (LEfSe) method revealed some of the specific microbial groups associated with clubroot disease suppression. At the genus level, *Botryotrichum*, *Chaetomium*, and *Acremonium* were more abundant in suppressive soils than in conducive soils. *Acremonium.* sp. was reported to exert biocontrol activity against plant pathogens such as *Aspergillus flavus*, *Fusarium verticillioides*, and *Meloidogyne incognita* (Donald et al., 2005; Yao et al., 2015). Indeed, *A. alternatum* has been developed for the control of clubroot (Jäschke et al., 2010). *Chaetomium* spp. belong to Ascomycota and have been reported as antagonists against several plant pathogens (Dhingra et al., 2003; Aggarwal et al., 2004; Park et al., 2005). Many species of *Chaetomium* with the potential to be biological control agents suppress the growth of bacteria and fungi through competition (for substrate and nutrients), mycoparasitism, antibiosis, or various combinations of these (Zhang and Yang, 2007). Interestingly, *Verticillium* was the most dominant genus in conducive soil. *Verticillium* is the pathogenic genus in the soil of *Verticillium* wilt-infected plants and can also infect cabbage (Dumin et al., 2021). Higher abundances of *RB41* and *Lysobacter* were found in suppressive soils. A recent report found that *RB41* plays an important role in controlling the carbon cycle (Stone et al., 2021). *Lysobacter* is a gram-negative bacterium widely distributed in diverse ecosystems, including soil and rhizosphere, and can promote plant growth and control soil-borne diseases (Expósito et al., 2015). Therefore, we infer that these beneficial bacteria are positively correlated with soil quality and plant health.

### 4.4 The fungal network was more complex in suppressive soil than in conducive soil

Microbial interactions are a vital part of the soil microbiome (Zhang et al., 2014), and the interactions among different microbial groups are important for determining ecosystem functioning/stability (Zhou et al., 2011); thus, perhaps a more integrated, complex network is more difficult for a pathogen or other intruder to enter. Many studies have demonstrated that microbial networks in healthy soils are more complex and stable than those in diseased soils (Yang et al., 2017). In this study, the suppressive soil showed a higher number of links than the conducive soil for fungal networks, whereas the conducive soil had higher link numbers than the suppressive soil for bacterial networks. This result is in agreement with a previous study (Xiong et al., 2017) and indicated that the fungal network of suppressive soils was more complex and stable than that of conducive soils. We also observed that the nodes of Ascomycota were the most numerous in the fungal network, which may be related to its high relative abundance in soils.

Furthermore, network analysis provides characterization not only of taxa with direct associations with important outcomes such as disease suppression but also of taxa with indirect associations via their association with other key taxa (Poudel et al., 2016). Connectors are essential microorganisms in the microbial network (Qi et al., 2019). More connectors possibly made more frequent exchanges of materials and information among the bacterial species in the conducive network than in the suppressive network. This result was also in agreement with a previous study in which the diseased soil had more connectors than the healthy soil (Jia et al., 2022). Indeed, we found that several potential beneficial fungi, such as *Botryotrichum*, *Chaetomium*, and *Acremonium*, were enriched in the suppressive soil, which may be the core taxa of the fungal network. *Acidibacter*, *Mitsuaria*, and *Phycicoccus* were enriched in the conducive soil, and they are often rare in agricultural soils. Therefore, we speculate that the host plant might selectively regulate the community abundance of some species under pathogen stress.

## 5 Conclusion

We found that the soil abiotic and biotic factors were distinct between clubroot-suppressive and clubroot-conducive soils. Specifically, the pH value and calcium content in the suppressive soils were higher than those in the conducive soils. The suppressive soils harbored higher fungal diversity and bacterial abundance than the conducive soils. Microorganisms beneficial to plants such as *Botryotrichum* and *Chaetomium* were more abundant in suppressive soils. The suppressive soil fungal network was more complex and stable and contained more interacting microbial species than the conducive soil fungal network. To demonstrate the inhibitory effect of microorganisms on clubroot, further studies identifying hyperdominant taxon isolates with effective clubroot disease suppression ability and revealing their interactions may open new avenues for the development of informed biocontrol strategies.

## Data availability statement

The datasets presented in this study can be found in online repositories. The names of the repository/repositories and accession number(s) can be found below: https://www.ncbi.nlm.nih.gov/genbank/, PRJNA982587.

## Author contributions

HK: Conceptualization, Writing – original draft. ZL: Conceptualization, Data curation, Writing – review and editing, Supervision. YS: Writing – review and editing. XX: Writing – review and editing. XY: Writing – review and editing. LL: Writing – review and editing. TF: Writing – review and editing. BL: Conceptualization, Writing – review and editing. AC: Conceptualization, Writing – review and editing.

## Footnotes

1
http://www.arb-silva.de

2
http://unite.ut.ee/index.php

3
http://www.mothur.org/wiki/Classify.seqs

4
http://ieg2.ou.edu/MENA/main.cgi
