# Supplementary material for: The occurrence of clubroot in cruciferous crops correlates with the chemical and microbial characteristics of soils
Source: Front Microbiol. 2024 Jan 8;14:1293360. doi: 10.3389/fmicb.2023.1293360 (PMC10800485; doi:10.3389/fmicb.2023.1293360)
Supplement: Supplementary file 1 [file Data_Sheet_1.docx]

Supplementary Material

# Supplementary Figures and Tables

## Supplementary Figures


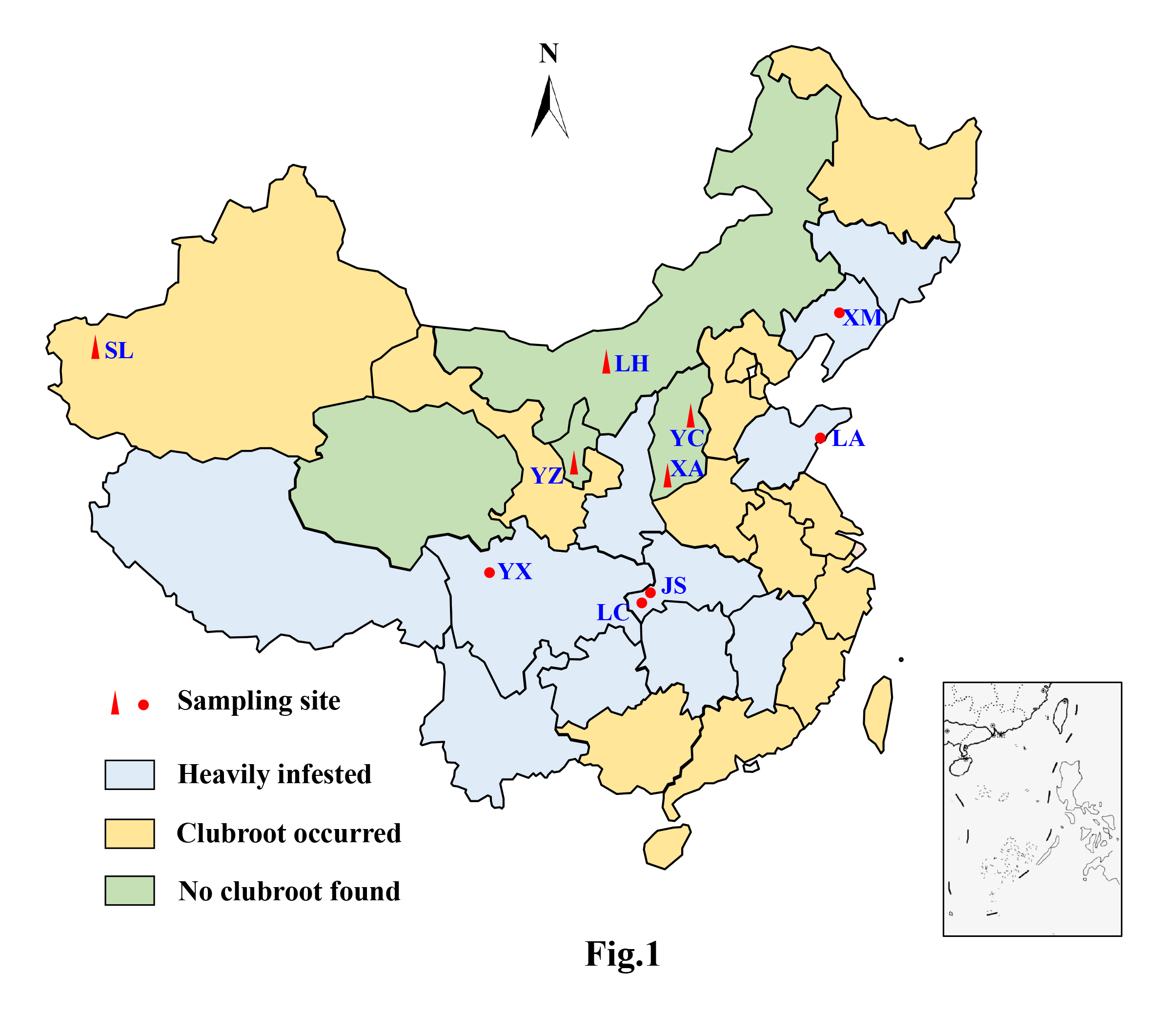


**Supplementary Figure 1.** **The distribution of clubroot-infested provinces in China and sampling sites.** The fields in XM, YX, LA, JS, and LC sites showed high incidence of clubroot. No clubroot was found in YC, XA, YZ, LH, and SL sites. XM, Xinmin City, Liaoning Province; LA, Licang District, Shandong Province; LC, Lichuan City, Hubei Province; JS, Jianshi County, Hubei Province; YX, Youxian District, Sichuan Province; YC, Yuci District, Shānxi Province; XA, Xia County, Shānxi Province; YZ, Yuanzhou District, Ningxia Hui Autonomous Region; SL, Shule County, Xinjiang Uygur Autonomous Region; LH, Linhe District, Inner Mongolia Autonomous Region. For each site, 3 random subplots (approximately 60 m^2^) were chosen, and soil samples from approximately 10 healthy (suppressive soil) or 10 clubroot-diseased (conducive soil) plants from each subplot were collected using the checkerboard sampling method during August 2020. Briefly, each subplot was divided into 10 areas and rhizosphere soil of plant in the central point of each area was collected (Niu et al., 2017). The 10 soil samples from each subplot were mixed to form one composite sample. The composite samples were placed into sterile bags and transported to the laboratory at 0℃. Samples were then divided into two subsamples: one portion (50 g) was stored at -80℃ for DNA extraction, and another (300 g) was used for chemical property analysis after thorough homogenization through a 2-mm sieve and air-drying. The soil (200 kg) used for the pot experiments was collected with a shovel from each subplot and thoroughly mixed for each site.

| **A**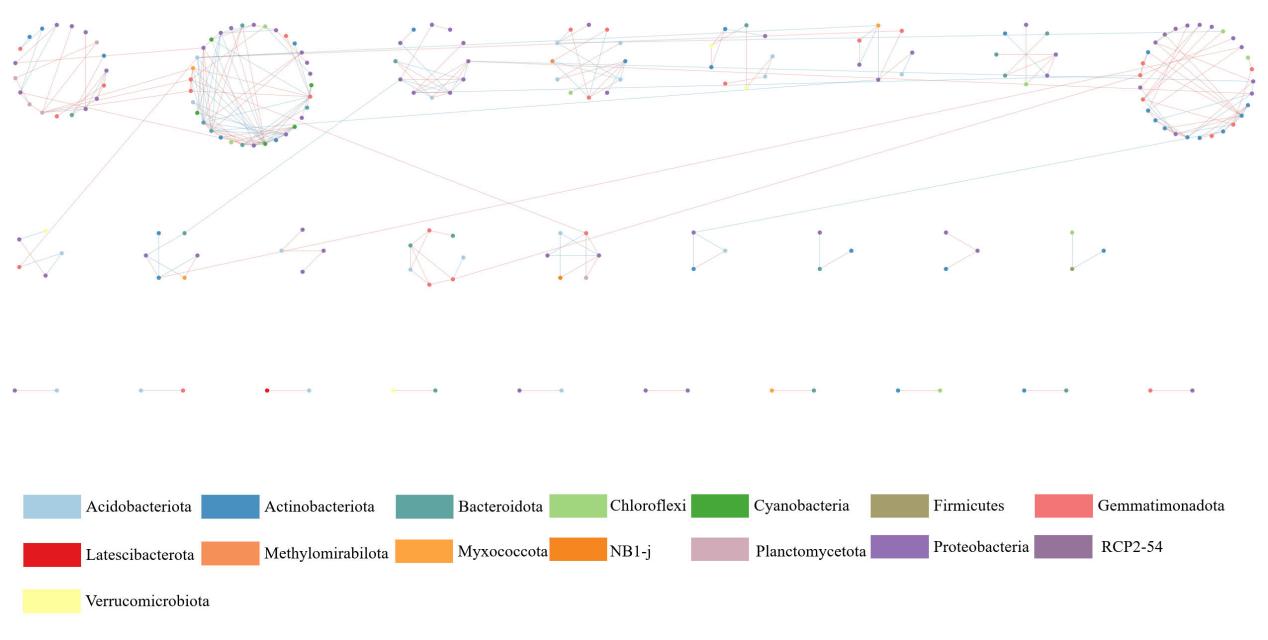 |
| --- |
| **B**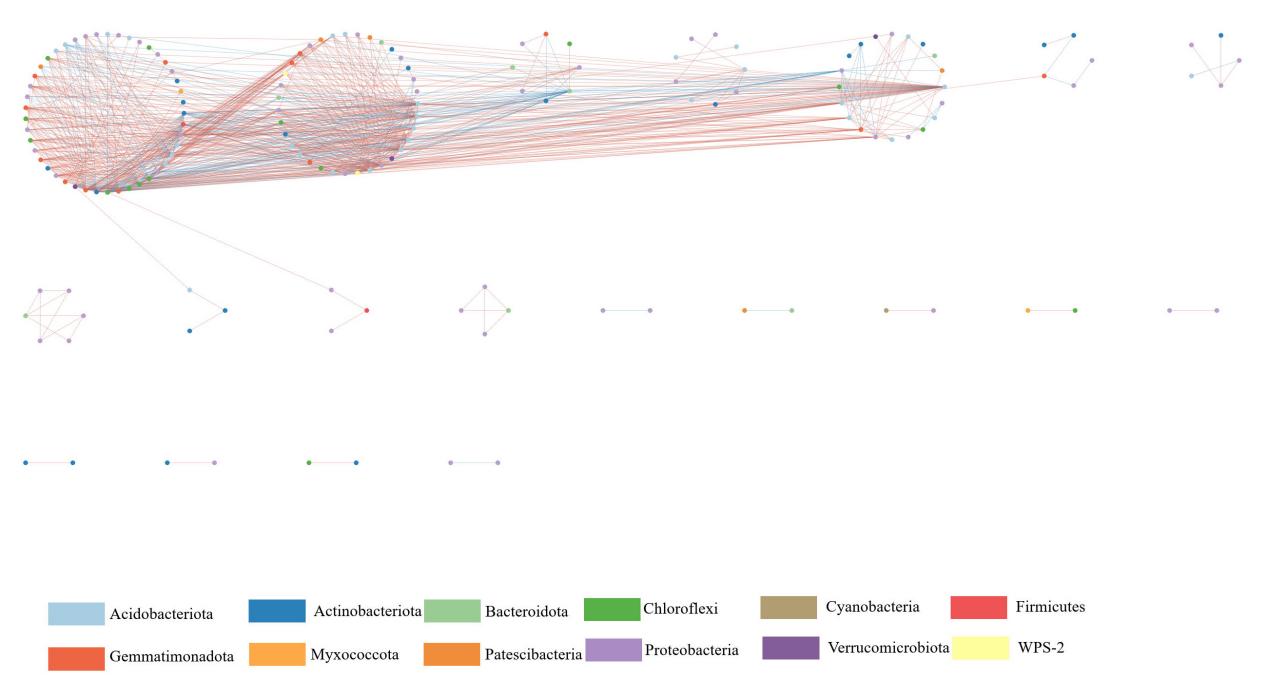 |
| **Supplementary Figure 2.** Network analyses of soil bacterial communities in suppressive (A) and conducive (B) soil. Nodes of different colors belong to different bacterial phyla. Blue edges represent negative interactions between nodes. Red edges represent positive interactions. |
| 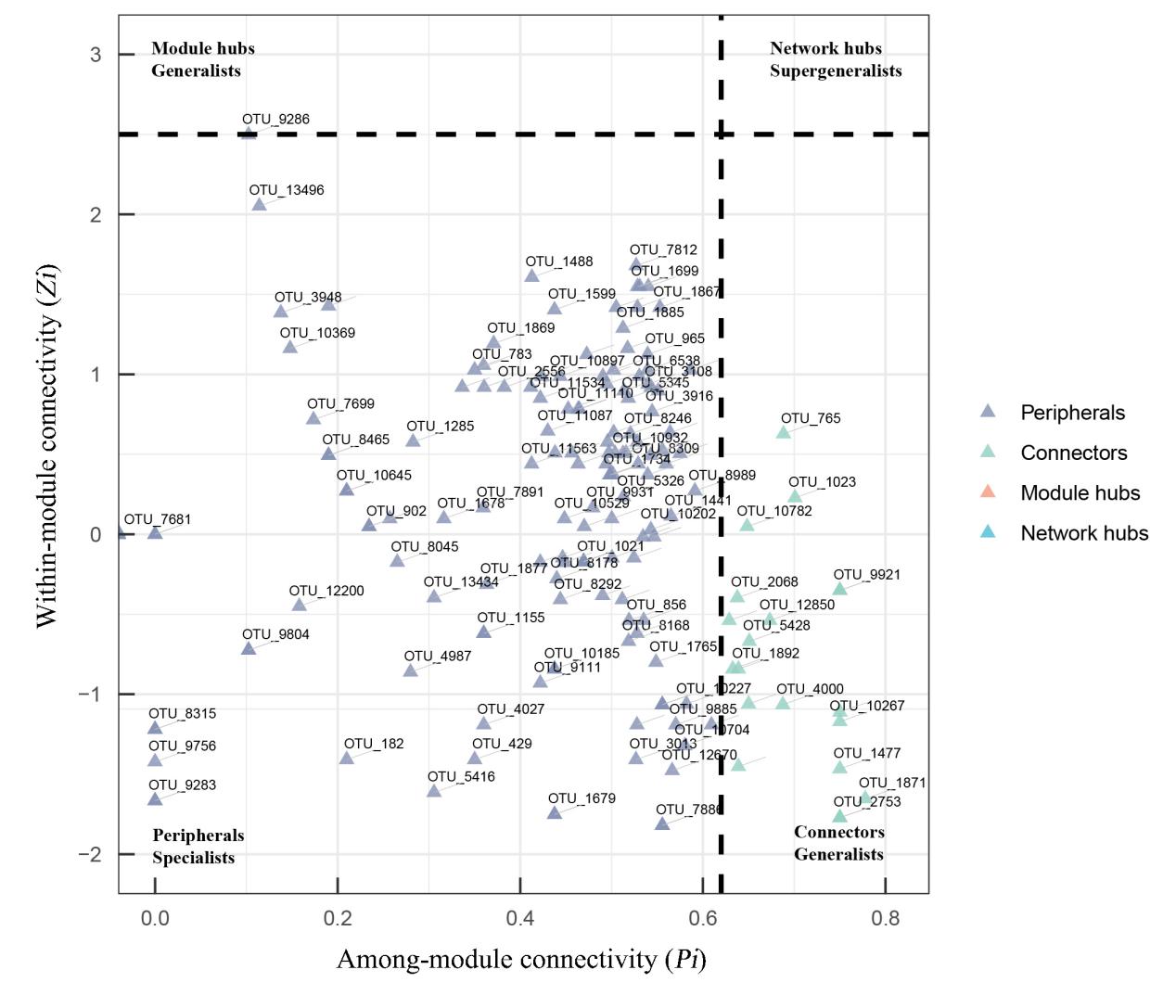 |
| **Supplementary Figure 3.** Zi-Pi plots showing the distribution of OTUs based on their topological roles in bacterial networks. The threshold values of Zi and Pi for categorizing OTUs are 2.5 and 0.62, respectively. Nodes are defined as peripherals (Pi ≤ 0.62, Zi ≤ 2.5), module hubs (Pi ≤ 0.62, Zi > 2.5), connectors (Pi > 0.62, Zi ≤ 2.5) and network hubs (Pi > 0.62, Zi > 2.5). |

## Supplementary Tables

**Supplementary Table 1.** Topological roles of top 200 nodes (OTUs) in fungal and bacteria networks of suppressive and conducive soils.

| Fungal network | | | | Bacteria network | | | |
| --- | --- | --- | --- | --- | --- | --- | --- |
| Nodes id | Suppressive | Conducive | Phylum | Nodes id | Suppressive | Conducive | Phylum |
| OTU_1278 | Connectors | Connectors | Ascomycota | OTU_10229 | No | Connectors | Proteobacteria |
| OTU_1515 | Connectors | Connectors | Ascomycota | OTU_1140 | No | Connectors | Proteobacteria |
| OTU_1733 | Connectors | Connectors | Ascomycota | OTU_11556 | Connectors | Connectors | Proteobacteria |
| OTU_1973 | Connectors | Connectors | Ascomycota | OTU_11675 | Connectors | Connectors | Proteobacteria |
| OTU_2031 | Connectors | Connectors | Ascomycota | OTU_12737 | Connectors | Connectors | Proteobacteria |
| OTU_2101 | Connectors | No | Ascomycota | OTU_1313 | No | Connectors | Proteobacteria |
| OTU_2150 | Connectors | Connectors | Ascomycota | OTU_1477 | No | Connectors | Proteobacteria |
| OTU_2328 | Connectors | Connectors | Ascomycota | OTU_1678 | Connectors | Connectors | Acidobacteriota |
| OTU_2453 | Connectors | Connectors | Ascomycota | OTU_4027 | Connectors | Connectors | Acidobacteriota |
| OTU_2546 | Connectors | Connectors | Ascomycota | OTU_5326 | Connectors | Connectors | Bacteroidota |
| OTU_2824 | Connectors | Connectors | Ascomycota | OTU_5414 | Connectors | Connectors | Bacteroidota |
| OTU_3058 | Connectors | Connectors | Ascomycota | OTU_7005 | No | Connectors | Gemmatimonadota |
| OTU_3530 | Connectors | Connectors | Mortierellomycota | OTU_765 | No | Connectors | Proteobacteria |
| OTU_3641 | Connectors | Connectors | Ascomycota | OTU_8313 | Connectors | Connectors | Actinobacteriota |
| OTU_3841 | Connectors | No | Ascomycota | OTU_8315 | Connectors | Connectors | Actinobacteriota |
| OTU_403 | Connectors | No | Olpidiomycota | OTU_8382 | Connectors | Connectors | Actinobacteriota |
| OTU_4103 | Connectors | Connectors | Ascomycota | OTU_9010 | No | Connectors | Chloroflexi |
| OTU_4194 | No | Connectors | Mortierellomycota | OTU_9886 | Connectors | Connectors | Planctomycetota |
| OTU_4203 | Connectors | Connectors | Ascomycota | OTU_9921 | Connectors | Connectors | Chloroflexi |
| OTU_471 | Connectors | No | Chytridiomycota | OTU_9931 | Connectors | Connectors | Proteobacteri |
| OTU_4714 | No | Connectors | Basidiomycota | The others 180 OTUs | Peripherals | Peripherals | —— |
| OTU_570 | Connectors | Connectors | Basidiomycota |  |  |  |  |
| OTU_955 | Connectors | Connectors | Ascomycota |  |  |  |  |
| The others 177 OTUs | Peripherals | Peripherals | —— |  |  |  |  |

No, the relative abundance of the node was 0%.
